# The Treatment of Rheumatic Fever

**Published:** 1907-09-28

**Authors:** Dyce Duckworth

**Affiliations:** Consulting Physician to St. Bartholomew's Hospital; Senior Physician to the Seamen's Hospital, Greenwich


### Hospital Clinics.

### THE TREATMENT OF RHEUMATIC FEVER.

By Sir DYCE DUCKWORTH, M.D., LLD., F.R.C.P., Consulting Physician to St. Bartholomew's Hospital; Senior Physician to the Seamen's Hospital, Greenwich.

A Clinical Lecture at the London School of Clinical Medicine, Greenwich.

Gentlemen,—I propose to discuss to-day the treatment of one of the commonest and most serious diseases with which we meet in this country. Acute rheumatism, or rheumatic fever, abounds mostly in temperate zones, is rare in high altitudes, but is more frequently met with in high, dry and exposed localities.* As you are aware, the term "rheumatism" is greatly abused, and is applied by people in all ranks of life to pains of many kinds.

For us the term has now more than ever a specific meaning, since our present knowledge, which has been remarkably extended within the last ten years, leads us to regard it as a distinct infective malady or toxæmia, due to specific and particulate germinal matter.

This new conception, arrived at by laborious and careful investigations, largely contributed to by Doctors Poynton and Paine in this country, and by several eminent Continental authorities, at once sweeps away all the older theories of the etiology of rheumatism. There still remain difficulties in determining the exact relations of this disease to other varieties of chronic rheumatism, but time will surely clear them up.

In the meantime we have a clear conception of the malady we are considering to-day, and while we regard its toxin as exogenous in character, we maintain the view that the toxic elements of gout, so often confounded with those of rheumatism, are of endogenous character—in other words, that the gouty patient manufactures his own toxins.

We note, in passing, the liability to true rheumatism in early life, its greater prevalence in the female sex, and the incidence on the latter of many of the irregular forms of the disease. We recognise the provoking agency of chill after exertion and exposure to rapid changes of temperature, of the debility induced by hyperlactation, and sometimes we see an epidemic character of incidence.

We make an arbitrary division into acute and subacute forms. In the latter we may find no fever, and may be misled in regarding such cases as unimportant. As a matter of fact, these mild cases may be as dangerous as more obviously severe ones. Hence it often happens that plain indications of past rheumatic disease are met with in patients who tell of no previous history of it. So I venture at once on this caution: Be always concerned about mild attacks of rheumatism in young patients, and do not make light of them.

There is a short incubation period. A sore throat is a common antecedent of pains and arthritis. This may pass away before the joints are involved. It is of more importance than is commonly believed, for there is good reason to think that this angina is significant of the channel of entry into the system of the specific poison. Next, we find flying pains in the muscles and joints, the larger of the latter being most involved. We do not meet with rigors, or very rarely. There is no symmetry in the arthritis, and the process may partly subside in one joint, and involve others for some days. Sweating is a marked symptom, and a peculiar sour, rank odour of the sweat is noticeable. The expression is anxious, decubitus dorsal, and no movements are made that can be avoided. The tongue is commonly coated, and the bowels are confined. The temperature is often about 102° F. at this stage. The pulse is full and soft. In children the temperature may be almost normal sometimes and the joints little involved; or there may be mere tenderness of the limbs, which is not seldom regarded by parents as "growing pains." There may be scanty urine, loaded with lithates, and sometimes a trace of albumin. The blood shows an increase of leucocytes.

What about the heart at this stage? You know that you have to examine it carefully each day, and if any of its structures are to be involved you may or may not know that this occurrence is to be expected at any time before the eighth day of the disease. If this term is overpassed it is not likely that any form of carditis will occur. Too often, alas, we find early some indication of endocarditis, usually involving the mitral curtains and giving rise to a systolic murmur. Again, we may note the friction murmur of pericarditis, commonly at the left margin of the sternum. This may be preliminary to some effusion into the sac of the pericardium, and if this occurs we are in face of a very grave condition. Any more severe complications, such as hyperpyrexia, cerebral symptoms, or coma, are now of rare occurrence under modern methods of treatment. Yet we must be prepared to meet with them occasionally.

The effects of rheumatic infection are not to be regarded as productive only of arthritis and some variety of carditis. We now recognise rheumatic disease of the fauces, of the skin, and of the brain and its membranes, some of these phases being antecedent to, or associated with, arthritis or carditis, as, for example, angina faucium, varieties of erythema, and chorea. We may meet with abarticular and apyrexial rheumatism, both of serious importance to note, because often overlooked or unrealised; important, too, because an associated endocarditis may be unnoticed and not sought for.

Without doubt the cardiac complications of rheumatism are the most grave. My reflections on the majority of cases of acute rheumatism which I have witnessed in a long hospital career leave a very sad impression on my mind as to the issues of the cardiac damage induced by it. It thus behoves us to do all in our power to avert, or to secure as sound a recovery as possible from, this condition.

* Collective Investigation Statistics Committee.

I have had experience of many varieties of special treatment which have been in vogue since my student days, and have watched the results of the calomel and opium method, the alkaline method, the employment of lemon juice in full amount, the blistering method, the salicin and salicylate treatment.

Each of these was regarded in its day as an advance in meeting pain and averting cardiac damage. Certainly nothing is more striking than the effect of sodium salicylate in affording ease to the patient. Yet it is still found that the infecting toxin is apt to alight upon the endocardium, and to work out its mischief there and in the other textures of the heart. It is still the exception to find a case of well-marked acute rheumatism running its course without involving the heart. Such cases, happily, do occur. Even in these we may find murmurs at the base of the heart which are proved to be inorganic, and due either to enfeeblement of the myocardium or to some degree of anæmia. The toxin of rheumatism is always responsible for a measure of the latter. These murmurs pass away on recovery.

At the present time we know no better method of treatment of acute rheumatism than that by pure sodium salicylate in doses of ten to fifteen grains every four hours for the first two days, and at longer intervals when the pain is subdued. The joints which are involved should be painted with belladonna liniment and wrapped in cotton wool. Nurses must not be too assiduous in washing such patients, only limb by limb should any necessary cleansing be carried out. All unnecessary movement is to be avoided, and the circulation thus kept quiet.

If valvular murmurs or pericardial friction be detected, it is advisable to put a small cantharidine blister over the site, and others may be applied from time to time over the præcordia. This particular treatment has been specially recommended by Dr. Caton, of Liverpool, in recent years, but it has long been a practice in St. Bartholomew's and other hospitals here, and has much to commend it.

Sometimes salicylate treatment is ill-borne by the patient, producing sickness and deafness. You may then substitute potassium iodide and citrate with cinchona, or quinine with advantage. At the outset it is well to give a calomel and colocynth purge, and then leave all aperients alone for a few days.

The diet is important. It should be entirely of milk and farinaceous matter. No beef-tea or animal food, so-called, is to be given. Whey, malt extract, and some alkaline water may be taken. I am familiar with cases retarded in their progress by beef-tea, soups, eggs, and strong food of that kind, which have rapidly yielded to the dietary I have recommended. In cases which are slow to improve under salicylate or other treatment benefit may be secured by small doses of calomel given at night, repeated two or three times, the diet being strictly of milk and farinaceous food.

The point that I now wish to lay stress upon in the treatment of cases where there are cardiac complications is the supreme importance of enjoining absolute rest in bed for a long period after the pains and febrile condition have passed off. The patient then commonly regards himself as cured, and is ready to get up and complete his convalescence in the ordinary manner. His friends are of the same opinion. I assume that at this stage the diet has been gradually improved, and that after allowing fish and a little meat, there are plain indications of general improvement. Yet the patient must, for his ultimate benefit, and in order to secure as sound a recovery as possible, remain recumbent in bed. I grant you this is not easy to enforce in the face of the relief already gained, the objections of relations, and possibly the claims of duty and occupation. When there are signs of valvular damage, with or without incompetence, consider what they signify. The process is that of a slowly healing endocarditis, proceeding to involution and cicatrisation. It is the last morbid result of the disease, undergoing gradual reduction, and in a part which is never at rest day or night. Is it wise to add to this unrest by permitting the patient to behave like an ordinary convalescent? Does not common sense, guided by accurate pathological knowledge, distinctly urge the extremest quietude and repose to allow efficient and complete healing to proceed in the damaged curtains? Yet the rules of some hospitals render it imperative to discharge such a patient after some absurdly prescribed limit of time. Now this necessary and prolonged rest may involve a stay in bed for some weeks. I am of opinion that many patients leave their wards in various hospitals before they are as soundly well as they might be, and this is a cruel proceeding. Some institutions make boast of the vast number of patients they minister to. It were better to treat fewer more efficiently, and for longer periods. What may we look for if we succeed in enforcing this long stay on the part of patients with rheumatic fever? I will tell you. Daily examination of the heart indicates, under this rest-cure, a distinct diminution of the damage done to the affected valve, a fading of the murmur, and a lessening of the temporary hypertrophy of the left ventricle. After two or three weeks we may find the murmur gradually disappearing, and the tone of the heart sounds improving.

During this time it is important to continue the application of small cantharadine blisters over different portions of the præcordia. These are of distinct value, and they also indicate to the patient that he still needs treatment. I do not declare, by any means, that you are to look for a good result in every case, for with your best efforts the damage may remain long in evidence, and may never be recovered from. Yet we meet with cases in after years where the signs of mischief have ultimately passed away, and left the heart in a very satisfactory condition.

In every case, however, I believe it to be our duty to secure this long rest in order to do our best for the patient. I generally give, during this period, quinine and iron, in the form of the syrup of phosphates of quinine, iron and strychnia. The patient must have the bowels and bladder relieved in bed, and must not rise for any purpose. You will meet with strong objection from the patient and his relatives to this seemingly unnecessary and prolonged confinement, but you must overcome this, and explain the importance of obedience to your orders.

